# The Use of Salicylic Acid in the Household

**Published:** 1877-05

**Authors:** 


### ARTICLE VII.

### *The Use of Salicylic Acid in the Household.*

BY DR. VON HEYDEN.

1. *Raw Meat.*—It frequently happens, especially in warm weather, that meat, particularly such as contains easily decomposed fat and blood, (tongues, etc.,) although otherwise irreproachable, upon closer examination or upon boiling, gives off a disagreeable smell. This may easily be removed either by laying the meat, before cooking, in lukewarm water containing half to one gram of salicylic acid to the litre, or by throwing some small crystals of acid into the water during the boiling.

When it is desired to preserve meat for some days, it is recommended to lay it in a solution of salicylic acid in water, half to one gram to the litre; or to rub lightly salicylic acid in the meat, especially the bones and fat parts. The preservation, as the cleaning for the dressing, is done in the usual way.

Although meat treated with salicylic acid loses its red color on the exterior, it undergoes no change internally. Moreover, it becomes tender with less boiling.

2. *Milk.*—Pure cow's milk, to which dry salicylic acid (not in aqueous solution) has been added in the proportion of half to one gram to the litre, curdles at the ordinary temperature after about thirty-six hours, retaining its properties, the cream separating and yielding butter perfectly.

3. *Butter* kneaded with water containing half to one gram of salicylic acid to the litre, or packed in cloths saturated in such a solution, remains good longer than usual. Butter that has already become rancid can be improved by careful washing with aqueous solution of salicylic acid (two to three grams to the litre) and afterwards rinsing with pure water.

4. *Preserved fruits* (cherries, currents, raspberries, plums, apricots, peaches) may be prepared advantageously, by placing layers of fruit and sugar alternately, without water, in a not very wide-mouthed pickle bottle, strewing over them a pinch of crystalized salicylic acid (about half a gram to a kilo of contents), closing the jar with parchment paper that has been steeped in solution of salicylic acid, and boiling the bottles in the ordinary way in a water-bath. Bilberries are best boiled without sugar, allowed to cool, filled into a narrow-mouthed flask, some crystalized salicylic acid strewn over, corked, etc. Fruit thus preserved has been kept in excellent condition during two seasons. Another method is to lay over the surface of fruit preserved in bottles a closely-fitting piece of blotting paper that has been steeped in a strong solution of salicylic acid in rum. Preserved gherkins may be similarly treated. For those preserved in vinegar and sugar (*Essiggurken*) the salicylic acid is boiled with the vinegar, and when boiled poured over the gherkins. For salt gherkins (*sauer gurken*) the acid, half to one gram to the litre, is added during the boiling; in other respects the preparation is as usual.

5. *Preserved Vegetables* and similar articles may also have a small quantity of crystalized salicylic acid added.

6. *Fumigations.* - Dry salicylic acid, volatnlized from a hot plate, purifies the air and perfectly disinfects the walls of a closed room.

7. *Vessels*, *Corks*, etc., to which a disagreeable smell or taste attaches, are thoroughly purified by washing in solution of salicylic acid.

The solutions of salicylic acid for the above purpose are best prepared by rapidly boiling the acid in water, in the proportion of from one to three grams to the litre, and leaving to cool. Any excess that then separates is fit for fresh use; or if stirred up and used in suspension causes a corresponding increase in the action of the solution.—*The Pharmacist.*

